# Chewing ability and associated factors in older adults in Germany. Results from GEDA 2019/2020-EHIS

**DOI:** 10.1186/s12903-023-03736-y

**Published:** 2023-12-09

**Authors:** Laura Krause, Stefanie Seeling, Anja Schienkiewitz, Judith Fuchs, Pantelis Petrakakis

**Affiliations:** 1 Department of Epidemiology and Health Monitoring, Robert Koch Institute, General-Pape-Str. 62–66, Berlin, 12101 Germany; 2 Federal Association of Dentists of the Public Health Service, Düsseldorf, Germany

**Keywords:** Oral health, Dental health, Oral impairments, Health status, Health behavior, Health care utilization

## Abstract

**Background:**

Oral well-being is an important component of general well-being and quality of life, as it is greatly influenced by the ability to chew and speak, and thus by central factors of social interaction. Because quality of life and participation are important factors for health in older age, the aim of this article was to examine the chewing ability, including associated factors, for the older population in Germany on the basis of a nationally representative sample.

**Methods:**

Database is the German Health Update (GEDA 2019/2020-EHIS), a population based cross-sectional survey of the Robert Koch Institute. In the telephone interview, participants aged 55 years and older were asked: “Do you have difficulty biting and chewing on hard foods such as a firm apple? Would you say ‘no difficulty’, ‘some difficulty’, ‘a lot of difficulty’ or ‘cannot do at all/ unable to do’?” Prevalences and multivariate prevalence ratios (PR) were calculated with 95% confidence intervals (95% CI) from log-Poisson regressions. Sociodemographic, health-, behavioral- and care-related characteristics were investigated as associated factors.

**Results:**

The analyses were based on data from 12,944 participants (7,079 women, 5,865 men). The proportion of people with reduced chewing ability was 20.0%; 14.5% had minor difficulty, 5.5% had major difficulty. There were no differences between women and men. The most important associated factors for reduced chewing ability were old age (PR 1.8, 95% CI 1.5–2.1), low socioeconomic status (PR 2.0, 95% CI 1.7–2.5), limitations to usual activities due to health problems (PR 1.9, 1.6–2.2), depressive symptoms (PR 1.7, 1.5–2.1), daily smoking (PR 1.6, 95% CI 1.3–1.8), low dental utilization (PR 1.6, 95% CI 1.4–1.9), and perceived unmet needs for dental care (PR 1.7, 95% CI 1.5–2.1).

**Conclusions:**

One fifth of adults from 55 years of age reported reduced chewing ability. Thus, this is a very common functional limitation in older age. Reduced chewing ability was associated with almost all investigated characteristics. Therefore, its prevention requires a holistic view in the living environment and health care context of older people. Given that chewing ability influences quality of life and social participation, maintaining or improving chewing ability is important for healthy aging.

## Background

Oral health is an essential component of general health and of great importance for quality of life and well-being [1]. Oral diseases such as caries and periodontitis can lead to oral impairments such as tooth loss and poorly fitting dentures [2]. Oral impairments can in turn be associated with discomfort and functional limitations, such as chewing disability [2]. Chewing ability is a general term that refers to the ability to put food into the mouth and bite, chew, and swallow it [3]. Functional limitations can in turn affect dietary choices and nutritional intake and therefore have consequences for general health [4]. In addition, functional limitations may go along with disinterest in eating with others due to discomfort [2]. Therefore, chewing ability can influence quality of life and social participation and thus is a very important factor for health in older age [2].

The chewing ability of older adults has been studied internationally [5–26]. The results show that reduced chewing ability increases with age [5, 9, 15–17, 19, 23], and that adults with low education or low income are more likely to have reduced chewing ability than those with high education or high income [5, 16, 17, 23, 27]. Regarding gender differences, the available results are inconsistent, showing either no differences [7, 9, 11, 15] or that women are more frequently affected by reduced chewing ability than men [5, 10, 16, 23]. In addition, a variety of further associated factors for reduced chewing ability have been identified, such as tooth loss [7, 9, 15–21, 23], tooth ache [5, 16, 21, 23, 24], limitations to daily activities [5, 8, 10, 11, 17], cognitive impairment [6, 7, 10, 11, 17, 18], depression [5, 7, 10, 25], lower health-related and oral health-related quality of life [5, 8, 9, 13, 20, 24, 27, 28], underweight [8, 12], poorer nutritional status (e.g. preference for food of soft consistency, lower food variety, lower fruit and vegetable consumption) [7, 15, 22, 26], daily smoking [11, 14, 17], lower utilization of dental services [23, 26], unmet need for dental care [29], and lower self-care [5, 17].

The majority of the studies cited above on chewing ability and its associated factors were conducted in the Asian region [5–14, 30, 31], followed by South America [15–17], and Anglo-Saxon countries [20–23]. However, the number of studies available from Europe is limited [18, 19]. The objective of this article is to examine the chewing ability and its associated factors among older adults in Germany, addressing a gap in the existing research. Furthermore, this study investigates various characteristics as potential associated factors, including gender, age, socioeconomic status (SES), limitations to usual activities due to health problems, underweight, depressive symptoms, daily smoking, daily fruit and vegetable consumption, dental utilization, perceived unmet needs for dental care, and home care service utilization. By analyzing these factors, this publication aims to provide insights into the chewing ability of older adults in Germany and its relationship with the aforementioned characteristics.

## Methods

### Study design and sample

Database is the German Health Update (GEDA), which is a nationwide representative cross-sectional survey of the resident population in Germany [32]. GEDA is conducted by the Robert Koch Institute in Berlin as part of the population-based health monitoring on behalf of the German Federal Ministry of Health. Since 2008, different GEDA waves have been realized. The questionnaire of the European Health Interview Survey (EHIS) [33] has been fully integrated in the GEDA study since the 2014/2015 wave [34]. Database for this analysis is the GEDA wave that took place between April 2019 and September 2020 as a telephone survey using a computer assisted, fully structured interview (Computer Assisted Telephone Interview, CATI) [35]. In GEDA 2019/2020-EHIS, a total of 23,001 persons aged 15 years and older were interviewed. The response rate of 22.0% was calculated according to the standards of the American Association for Public Opinion Research (AAPOR, RR3) [35, 36]. The questionnaire of GEDA 2019/2020-EHIS consists of four sections on the following areas: health status, health care, health determinants as well as demographic and socioeconomic characteristics of the participants.

### Outcome variable

#### Chewing ability

Participants aged 55 years and older were asked: “Do you have difficulty biting and chewing on hard foods such as a firm apple? Would you say – ‘no difficulty’, ‘some difficulty’, ‘a lot of difficulty’ or ‘cannot do at all/unable to do’.” For the analyses, the four response options were regrouped into three categories: no difficulty, minor difficulty (‘some difficulty’) and major difficulty (‘a lot of difficulty’ and ‘cannot do at all/unable to do’). In a first step, the proportion of people who have minor or major difficulty is reported (minor and major difficulty separated). In a second step, the proportion of people who have any difficulty is shown (minor and major difficulty together). To investigate chewing ability in a more differentiated way, sociodemographic characteristics as well as health-, behavior- and care-related factors were used for stratification.

### Sociodemographic factors

#### Gender

GEDA 2019/2020-EHIS used gender identities to describe gender differences and allowed the respondents to indicate which gender they feel they belong to [37].

#### Age

For the analyses, the age groups were divided according to the recommendation of the World Health Organization (WHO) [38]: 55–64 years, 65–74 years, and 75 years and older.

#### Socioeconomic status (SES)

The SES of participants is based on a multidimensional additive index that includes information on educational level (CASMIN educational classification [39]), income situation, and occupational status [40, 41].

### Health-related factors

#### Limitations to usual activities due to health problems

Participants were asked: “Are you limited because of a health problem in activities people usually do? Would you say you are – ‘severely limited’, ‘limited but not severely’ or ‘not limited at all’?” Persons who answered that they are ‘severely limited’ or ‘limited but not severely’ were then asked: “Have you been limited for at least the past 6 months?” The response categories were “yes” and “no” [42]. Participants who answered that they were “severely limited” or “limited but not severely” in their usual activities for over 6 months are considered as limited due to health problems [43].

#### Underweight

There is no universally accepted definition of malnutrition in older age. In the guideline of the German Society for Nutritional Medicine (DGEM), malnutrition is defined as an unintentional noticeable weight loss (> 5% in three months or > 10% in six months) or a significantly reduced body mass (Body Mass Index, BMI < 20 kg/m^2^) [44]. In this definition, the DGEM refers to a group of people with an average age of at least 65 years. The mean age of the present study population is 69 years (min: 55 years, max: 99 years; Std. dev.: 9,07). In GEDA 2019/2020-EHIS, BMI can be calculated based on self-reported data on body weight and height. Body weight was assessed by the question: “How much do you weigh without clothes and shoes? Please state your body weight in kilograms.” Body height was ascertained by asking: “How tall are you when you are not wearing shoes?” The information was given in centimeters. Following the guideline of DGEM, underweight is defined in this article as a BMI < 20 kg/m^2^.

#### Depressive symptoms

A country-specific version of the internationally established 8-item Patient Health Questionnaire (PHQ-8) [45] was used to assess depressive symptoms [46]. The PHQ-8 comprises symptoms of a major depression during the last two weeks in line with the Diagnostic and Statistical Manual of Mental Disorders (DSM-IV, 4th edition [47]): depressed mood, diminished interest, significant weight loss or poor appetite, insomnia or hypersomnia, psychomotor agitation or retardation, fatigue or loss of energy, feelings of worthlessness or excessive or inappropriate guilt, diminished ability to think or concentrate. Each of these items was rated on a scale ranging from 0 (not at all), 1 (on individual days), 2 (more than half of the days) to 3 (nearly every day). Answers are summarized to a total score and a depressive symptomatology is assumed from a value of at least 10. While values between 10 and 14 indicate a ‘mild’ depressive symptomatology, values greater 14 point to a ‘moderate to severe’ depressive symptomatology [45].

### Behavior-related factors

#### Daily smoking

Participants were asked: “Do you smoke any tobacco products (excluding electronic cigarettes or similar electronic devices)?” The response categories were “yes, daily”, “yes, occasionally”, “no longer” and “I’ve never smoked”. For the analyses the last three categories were combined. Thus, a distinction can be made between daily and not daily tobacco smoking [48].

#### Daily fruit and vegetable consumption

Participants were asked: “How often do you eat fruit? Frozen, dried, canned, etc. fruits should be included. But any fruit juices should be excluded.” and “How often do you eat vegetables or salad? Frozen, dried, canned, etc. vegetables should be included. But any kind of vegetable juices or soups (warm and cold) should be excluded.” The response categories for each question were “once or more a day”, “4 to 6 times a week”, “1 to 3 times a week”, “less than once a week” and “never” [49]. In order to be able to indicate a daily fruit and vegetable consumption, the data were summarized and the 5-point response scale was dichotomized into “daily” vs. “not daily”. Persons who did not answer one of these two questions were excluded from the analyses.

### Care-related factors

#### Dental utilization

Participants were asked: “When was the last time you visited a dentist or orthodontist on your own behalf (that is, not while only accompanying a child, spouse, etc.)? Would you say – ‘less than 6 months’, ‘6 to less than 12 months’, ‘12 months or longer’ or ‘never’.” The first and the last two categories were combined for the analyses. In this way, the indicator of the 12-month prevalence of dental utilization (yes/no) is obtained [50].

#### Perceived unmet needs for dental care

Participants were asked: “Has it happened in the last 12 months that you needed one of the following examinations or treatments but could not afford them?” The answers options included: “dental or orthodontic examination or treatment”. The answer categories were “yes”, “no” and “no need”. Persons who had no need were excluded from the analyses [51].

#### Home care service utilization

Participants were asked: “In the past 12 months, have you yourself used or received any home care services?” Only services provided by professional health or social workers should be included. The response categories were “yes” and “no”.

### Statistical analysis

The results are presented as prevalences with 95% confidence intervals (95% CI) and stratified by sociodemographic as well as health-, behavior- and care-related factors. Multivariate log-Poisson regression models with dichotomized chewing ability as the dependent outcome variable were applied to determine whether the differences between groups are significant. Prevalence ratios (PR) with 95% CI were calculated as effect estimates for reduced chewing ability. Regression analyses for women and men combined were adjusted for gender, age, and SES, regression analyses for women and men separately for age and SES. Finally, a log-Poisson regression model with all stratification characteristics considered in this article was calculated (multivariate overall model). A significant difference between groups is assumed if the calculated p-value is < 0.05.

The analyses were carried out using a weighting factor to correct for deviations of the sample from the population structure. Design weighting was first carried out for the different selection probabilities (mobile and landline) [52]. This was followed by an adjustment to the official population figures based on age, sex, residential structure (Federal Statistical Office 2019), and education distribution (Microcensus 2017) [35]. All analyses were conducted with the survey procedures for complex samples of StataSE 17.0.

## Results

The analyses were based on information from 12.985 participants aged 55 years and older, including 7,086 women and 5,871 men (Table 1). 28 respondents provided a different gender identity to the one that they were assigned at birth or gave no information. Due to the limited number, these individuals were not considered in the gender stratified analyses. However, they remained in the total category.

**Table 1:** Characteristics of the study population

	%	n
**Total**	100	12.985
**Difficulty in biting and chewing on hard foods**		
No difficulty	80.0	10,994
Some difficulty	14.5	1,519
A lot of difficulty	3.4	312
Cannot do at all/unable to do	2.1	147
Missing values	−	13
**Gender**		
Women	53.9	5,871
Men	46.1	7,086
Missing values	−	28
**Age group**		
55–64 years	36.9	4,628
65–74 years	30.3	4,573
75 years and older	32.9	3,784
**Socioeconomic status**		
Low	22.1	1,210
Medium	60.3	7,049
High	17.5	4,693
Missing values	−	33
**Limitations to usual activities due to health problems**		
No	53.0	7,602
Yes	47.0	5,341
Missing values	−	42
**Underweight**		
No	95.7	12,260
Yes	4.3	567
Missing values	−	158
**Depressive symptoms**		
No	93.1	12,013
Yes	6.9	663
Missing values	−	309
**Daily smoking**		
No	83.5	11,156
Yes	16.5	1,669
Missing values	−	160
**Daily fruit and vegetable consumption**		
No	63.1	7,652
Yes	36.9	5,319
Missing values	−	14
**12-month prevalence of dental utilization**		
No	18.7	1,854
Yes	81.3	11,122
Missing values	−	9
**12-month prevalence of perceived unmet needs for dental care**		
No	93.4	9,917
Yes	6.6	579
Missing values	−	2,489
**12-month prevalence of home care service utilization**		
No	91.8	12,265
Yes	8.2	716
Missing values	−	4

Source: GEDA 2019/2020-EHIS

% = weighted percentages of respondents with complete variable information

n = unweighted numbers of respondents with valid information

Of the 12,985 participants, 12,972 had valid information on the outcome variable of chewing ability. The different numbers of missing data for the variables gender, age, SES, limitations to usual activities due to health problems, underweight, depressive symptoms, daily smoking, daily fruit and vegetable consumption, dental utilization, perceived unmet needs for dental care, and home care service utilization, resulted in different number of cases for each outcome (Table 1).

**Fig. 1:**
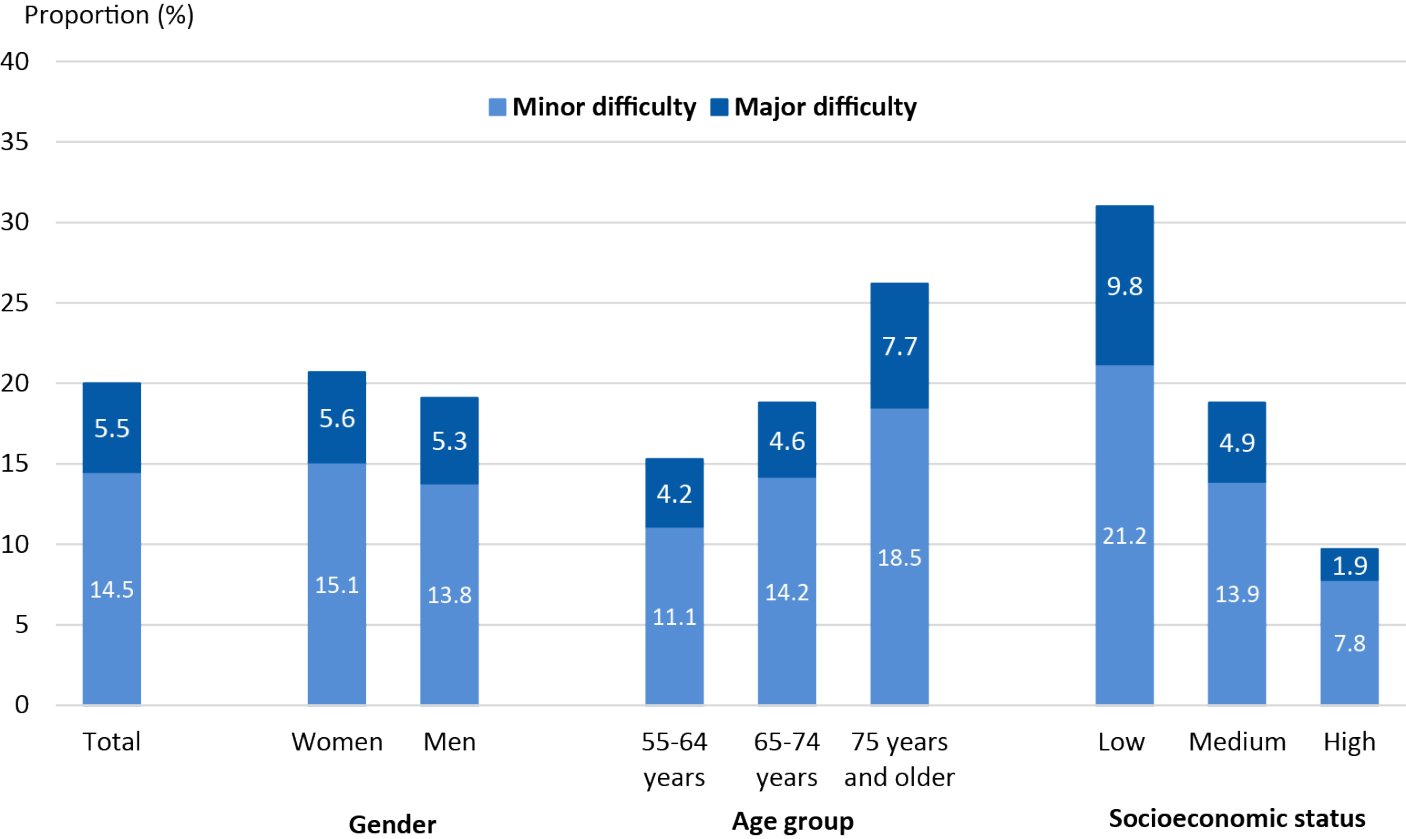
Reduced chewing ability^1^ according to sociodemographic factors in persons aged 55 years and older. ^1^Minor and major difficulty separated

Figure 1 shows that 20.0% of respondents aged 55 years and older had reduced chewing ability – 14.5% reported minor difficulty and 5.5% reported major difficulty.

### Sociodemographic factors

There were no statistically significant differences in chewing ability between women and men (Table 2). In addition, reduced chewing ability increased significantly with increasing age (p < 0.001): while in the age group 55 to 64 years about one in seven reported reduced chewing ability (15.3%), it was about one in four in the age group 75 years and older (26.3%). Moreover, the proportion of adults having reduced chewing ability increased with decreasing SES. Overall, the risk of reporting reduced chewing ability was increased by a factor of 1.9 in adults with medium SES, and by a factor of 3.1 in adults with low SES compared to those with high SES (p < 0.001). Here, adults with low and high SES differed particularly strong in terms of major difficulty (Figure 1): the proportion of people with major difficulty was five times higher among those with low than with high SES (9.8% and 1.9%).

**Table 2:** Reduced chewing ability^1^ according to sociodemographic factors in persons aged 55 years and older

	% (95% CI)^2^	PR (95% CI))^3^	p
**Total**	20.0 (18.8–21.2)	–	–
**Gender**			
Women	20.7 (19.1–21.0)	1.0 (0.9–1.1)	0.902
Men	19.1 (17.4–22.4)	ref.	–
**Age group**			
55–64 years	15.4 (13.7–17.3)	ref.	–
65–74 years	18.8 (16.9–20.9)	1.2 (1.1–1.4)	0.007
75 years and older	26.2 (23.9–28.7)	1.7 (1.4–1.9)	< 0.001
**Socioeconomic status**			
Low	31.0 (27.5–34.7)	3.1 (2.6–3.7)	< 0.001
Medium	18.8 (17.5–20.3)	1.9 (1.6–2.2)	< 0.001
High	9.7 (8.6–11.0)	ref.	–

PR = prevalence ratio, 95% CI = 95% confidence interval, ref. = reference group

^1^No difficulty vs. minor and major difficulty together, ^2^Proportions in percent (%), ^3^Results of multivariate log-Poisson regressions

### Health-related factors

Adults aged 55 years and older who had reduced chewing ability were more likely to have limitations in usual activities due to health problems and depressive symptoms compared to persons of the same age without such difficulties (Table 3). For all associations, the PR was slightly above 2 for women and men (p < 0.001). This was also true for the PR of underweight in men while the relationship in women was less pronounced (PR 1.3; p = 0.041).

**Table 3:** Reduced chewing ability^1^ according to health-related factors in persons aged 55 years and older

	Women			Men			Total		
	**%** ^2^ **(95% CI)**	**PR** ^3^ **(95% CI)**	**p**	**%** ^2^ **(95% CI)**	**PR** ^3^ **(95% CI)**	**p**	**%** ^2^ **(95% CI)**	**PR** ^3^ **(95% CI)**	**p**
**Limitations to usual activities due to health problems**									
No	12.4 (10.9–14.1)	ref.	–	11.3 (9.5–13.2)	ref.	–	11.9 (10.7–13.1)	ref.	–
Yes	29.5 (26.8–32.3)	2.2 (1.8–2.5)	< 0.001	28.4 (25.4–31.6)	2.2 (1.8–2.7)	< 0.001	29.0 (27.0-31.1)	2.2 (1.9–2.5)	< 0.001
**Underweight**									
No	20.1 (18.5–21.8)	ref.	–	18.4 (16.7–20.2)	ref.	–	19.3 (18.1–20.5)	ref.	–
Yes	27.2 (20.7–34.8)	1.3 (1.0-1.8)	0.041	46.3 (29.1–64.4)	2.2 (1.4–3.3)	< 0.001	31.3 (24.8–38.6)	1.6 (1.2-2.0)	< 0.001
**Depressive Symptoms**									
No	18.7 (17.1–20.3)	ref.	–	16.9 (15.3–18.7)	ref.	–	17.9 (16.7–19.1)	ref.	–
Yes	40.7 (33.6–48.2)	2.2 (1.8–2.7)	< 0.001	45.1 (35.1–55.4)	2.3 (1.8-3.0)	< 0.001	42.6 (36.6–48.8)	2.3 (1.9–2.7)	< 0.001

PR = prevalence ratio, 95% CI = 95% confidence interval, ref. = reference group

^1^No difficulty vs. minor and major difficulty together, ^2^Proportions in percent (%), ^3^Results of multivariate log-Poisson regressions

### Behavior-related factors

Compared to the reference group, adults aged 55 years and older with reduced chewing ability were more likely to smoke daily and to have non-daily fruit and vegetable consumption (Table 4). The association between reduced chewing ability and daily smoking was particularly strong in men: while every third man reported smoking daily in the group with reduced chewing ability (34.8%), it was only every sixth man in the group without such difficulties (15.5%). Overall, the risk of reduced chewing ability was increased by a factor of 2.4 in men who smoked daily compared to men who did not (p < 0.001).

**Table 4:** Reduced chewing ability^1^ according to behavior-related factors in persons aged 55 years and older

	Women			Men			Total		
	**%** ^2^ **(95% CI)**	**PR** ^3^ **(95% CI)**	**p**	**%** ^2^ **(95% CI)**	**PR** ^3^ **(95% CI)**	**p**	**%** ^2^ **(95% CI)**	**PR** ^3^ **(95% CI)**	**p**
**Daily smoking**									
No	19.6 (17.9–21.4)	ref.	–	15.5 (13.9–17.2)	ref.	–	17.8 (16.6–19.0)	ref.	–
Yes	26.4 (22.1–31.3)	1.6 (1.3-2.0)	< 0.001	34.8 (29.3–40.8)	2.4 (1.9–2.9)	< 0.001	30.7 (27.1–34.6)	2.0 (1.7–2.3)	< 0.001
**Daily fruit and vegetable consumption**									
Yes	17.8 (15.6–20.1)	ref.	–	16.5 (13.0-19.1)	ref.	–	17.4 (15.7–19.3)	ref.	–
No	23.2 (21.0-25.6)	1.3 (1.1–1.5)	0.002	20.1 (18.0-22.3)	1.1 (0.9–1.4)	0.368	21.5 (20.0-23.1)	1.2 (1.1–1.4)	0.003

PR = prevalence ratio, 95% CI = 95% confidence interval, ref. = reference group

^1^No difficulty vs. minor and major difficulty together, ^2^Proportions in percent (%), ^3^Results of multivariate log-Poisson regressions

### Care-related factors

Women and men aged 55 years and older who had reduced chewing ability were less likely to have visited a dental practice in the year prior to the survey and they were more likely to have unmet dental care needs; they were also more likely to use a home care service compared to those of the same age without difficulties (Table 5). For all care-related factors, the relation with reduced chewing ability was comparably strong (PR between 1.4 and 1.9; p < 0.001 in each case).

**Table 5:** Reduced chewing ability^1^ according to care-related factors in persons aged 55 years and older

	Women			Men			Total		
	**%** ^2^ **(95% CI)**	**PR** ^3^ **(95% CI)**	**p**	**%** ^2^ **(95% CI)**	**PR** ^3^ **(95% CI)**	**p**	**%** ^2^ **(95% CI)**	**PR** ^3^ **(95% CI)**	**P**
**12-month prevalence of dental utilization**									
Yes	18.3 (16.7–20.0)	ref.	─	15.5 (13.8–17.4)	ref.	–	17.1 (15.9–18.4)	ref.	–
No	32.5 (27.8–37.7)	1.5 (1.3–1.8)	< 0.001	32.9 (28.2–37.9)	1.9 (1.6–2.3)	< 0.001	32.7 (29.3–36.3)	1.7 (1.5–1.9)	< 0.001
**12-month prevalence of perceived unmet needs for dental care**									
No	19.3 (17.5–21.2)	ref.	─	17.8 (15.9–19.9)	ref.	─	18.6 (17.3–20.0)	ref.	–
Yes	36.4 (28.8–44.7)	1.8 (1.4–2.4)	0.001	37.3 (29.1–46.3)	1.9 (1.5–2.5)	< 0.001	36.8 (31.1–42.9)	1.9 (1.6–2.3)	< 0.001
**12-month prevalence of home care service utilization**									
No	18.9 (17.3–20.5)	ref.	─	18.1 (16.4–20.0)	ref.	–	18.5 (17.4–19.8)	ref.	–
Yes	38.7 (32.0-45.8)	1.7 (1.4–2.1)	< 0.001	32.5 (24.6–41.5)	1.4 (1.1–1.8)	0.033	36.3 (31.1–41.8)	1.6 (1.4–1.9)	< 0.001

PR = prevalence ratio, 95% CI = 95% confidence interval, ref. = reference group

^1^No difficulty vs. minor and major difficulty together, ^2^Proportions in percent (%), ^3^Results of multivariate log-Poisson regressions

### Multivariate overall model

When all stratification characteristics were included in one model, the most important associated factors of reduced chewing ability in adults from 55 of age were: old age (75 years and older, PR 1.8), low SES (PR 2.0), limitations to usual activities due to health problems (PR 1.9), depressive symptoms (PR 1.7), daily smoking (PR 1.6), low dental utilization (PR 1.6), and perceived unmet needs for dental care (PR 1.7). This applied to both women and men (Table 6).

**Table 6:** Reduced chewing ability^1^ according to all considered stratification characteristics

	Women		Men		Total	
	**PR (95% CI)** ^2^	**p**	**PR (95% CI)** ^2^	**p**	**PR (95% CI)** ^2^	**p**
**Gender**						
Women	–	–	–	–	ref.	–
Men	–	–	–	–	1.0 (0.9–1.1)	0.899
**Age group**						
55–64 years	ref.	–	ref.	–	ref.	–
65–74 years	1.3 (1.0-1.6)	0.035	1.4 (1.1–1.8)	0.002	1.3 (1.1–1.6)	< 0.001
75 years and older	1.7 (1.4–2.1)	< 0.001	1.9 (1.5–2.5)	< 0.001	1.8 (1.5–2.1)	< 0.001
**Socioeconomic status**						
Low	1.8 (1.3–2.4)	< 0.001	2.2 (1.7–2.9)	< 0.001	2.0 (1.7–2.5)	< 0.001
Medium	1.3 (1.1–1.7)	0.017	1.5 (1.2–1.9)	< 0.001	1.5 (1.2–1.7)	< 0.001
High	ref.	–	ref.	–	ref.	–
**Limitations to usual activities due to health problems**						
No	ref.	–	ref.	–	ref.	–
Yes	1.8 (1.5–2.2)	< 0.001	1.9 (1.5–2.4)	0.001	1.9 (1.6–2.2)	< 0.001
**Underweight**						
No	ref.	–	ref.	–	ref.	–
Yes	1.4 (1.0-1.9)	0.052	1.3 (0.9-2.0)	0.219	1.4 (1.1–1.8)	0.016
**Depressive symptoms**						
No	ref.	–	ref.	–	ref.	–
Yes	1.7 (1.4–2.2)	< 0.001	1.8 (1.4–2.3)	< 0.001	1.7 (1.5–2.1)	< 0.001
**Daily smoking**						
No	ref.	–	ref.	–	ref.	–
Yes	1.4 (1.1–1.7)	0.007	1.7 (1.4–2.2)	< 0.001	1.6 (1.3–1.8)	< 0.001
**Daily fruit and vegetable consumption**						
Yes	ref.	–	ref.	–	ref.	–
No	1.1 (0.9–1.3)	0.368	1.0 (0.8–1.2)	0.706	1.0 (0.9–1.2)	0.649
**12-month prevalence of dental utilization**						
Yes	ref.	–	ref.	–	ref.	–
No	1.4 (1.2–1.8)	0.001	1.8 (1.4–2.2)	< 0.001	1.6 (1.4–1.9)	< 0.001
**12-month prevalence of perceived unmet needs for dental care**						
No	ref.	–	ref.	–	ref.	–
Yes	1.7 (1.4–2.2)	< 0.001	1.8 (1.4–2.3)	< 0.001	1.7 (1.5–2.1)	< 0.001
**12-month prevalence of home care service utilization**						
No	ref.	–	ref.	–	ref.	–
Yes	1.1 (0.9–1.5)	0.383	1.1 (0.8–1.4)	0.619	1.1 (0.9–1.3)	0.342

PR = prevalence ratio, 95% CI = 95% confidence interval, ref. = reference group

^1^No difficulty vs. minor and major difficulty together, ^2^Results of multivariate log-Poisson regressions (multivariate overall model)

## Discussion

The results from GEDA 2019/2020-EHIS show that 20.0% of adults from 55 years of age reported reduced chewing ability. Of these, 14.5% had minor difficulty and 5.5% had major difficulty. The international studies cited in the introduction provide prevalences that deviate from the present results [5–26]. Reasons for this are, for example, differences in the considered age groups or differences in the operationalization of the indicator. The question used to determine chewing ability varied in the studies: The majority of them asked about *chewing* [5–11, 13–15, 17, 18, 20, 24], only a few, as in the present one, asked about *chewing and biting* [22, 29].

In the context of varying prevalences of oral health parameters between countries, socio-cultural aspects also play a role in influencing oral health, such as insufficient exposure to fluoride or difficult access to oral and dental care products [53]. Therefore, studies from Germany are particularly relevant for an interpretation of the present results. The German Oral Health Study from the Institute of German Dentists provides both survey data and clinical data on the oral health of the population in Germany [54]. In this study, data on the oral health of selected age groups are collected. According to the data from the fifth survey (2014), 31.3% of 65- to 74-year-olds had reduced chewing ability. In GEDA 2019/2020-EHIS, the corresponding figure is 18.8%. However, a direct comparison of the results is not possible, for example, due to the different question and answer categories. The question asked in the German Oral Health Study was “Do you have difficulty chewing solid food (e.g. fruit, bread, meat, etc.)?” with the response options “not at all”, “a little”, “partly”, “relatively strong” and “very strong/makes great difficulties” [54]. Even though the results are not directly comparable, both studies indicate a higher proportion of older adults who have reduced chewing ability. In a study examining the probability to bite and chew hard foods in older adults from 14 European countries, Germany was ranked 5th, i.e. in the upper midfield [55].

Factors associated with reduced chewing ability among women and men were old age, low SES, limitations to usual activities due to health problems, depressive symptoms, daily smoking, low dental utilization, and perceived unmet needs for dental care. Thus, the present results are in line with the above-mentioned international studies [5, 7–11, 14–17, 19, 23, 25, 26, 29]. The finding that more people are affected by reduced chewing ability with increasing age is supported by the fact that oral diseases and oral impairments, which in turn can lead to functional limitations such as chewing disability, occur more frequently with increasing age [56]. The social gradient observed in reduced chewing ability, which disadvantages individuals with low SES, is similarly evident in the occurrence of oral diseases and oral impairments [56]. Moreover, adults who smoke daily were more likely to have reduced chewing ability than non-daily smokers. Oral diseases and oral impairments are more common in smokers than in non-smokers, as smoking damages the oral cavity in many ways due to the pollutants contained in tobacco smoke [57]. People who did not visit a dental office in the year prior to the survey were, as expected, more likely to report reduced chewing ability than those with appropriate utilization. Regular dental visits can detect oral diseases at an early stage and suitable measures can be initiated to prevent oral impairments and functional limitations [58]. Beyond that, adults who stated that they needed dental care but could not afford it were more likely to have reduced chewing ability than those without such problems. Financial reasons are the most common cause why necessary dental treatments are not perceived [59]. In addition, the results illustrate a relationship between reduced chewing ability and limitations to usual activities due to health problems. In this context, one study indicated that older people who are still active tend to be more motivated to maintain their oral health and have less difficulty brushing their teeth and attending dental check-ups [7]. Additionally, older people who are still active are generally mentally fitter and thus better able to understand oral health-related information [7]. The results also suggest a relationship between reduced chewing ability and depressive symptoms as screened by the PHQ-8, which measures symptoms including depressed mood, decreased interest, fatigue, and loss of energy [45]. One possible explanation for this association could be that affected people are unable to take adequate care of their oral health due to their mental state [60]. Furthermore, studies show that chewing ability also influences food choices, which in turn can affect diet and body weight [7, 8, 22, 26]. In the present study, however, the association between reduced chewing ability and non-daily fruit and vegetable consumption and underweight, respectively, was significant only in the univariate but not in the multivariate model. The same applied to the association between reduced chewing ability and the utilization of home care services. In contrast, other studies suggest that reduced chewing ability is associated with lower self-care [5, 17]. In future, longitudinal studies focusing on the effect of the considered characteristics on chewing ability are required to show possible causal relationships [25].

The present analyses point to *prevention potentials* and *healthcare needs*. In order to develop tailor-made prevention measures, it is important to identify vulnerable groups. According to the results, especially people from 75 of age and those with low SES reported reduced chewing ability. In addition, the results show that daily smoking is associated with reduced chewing ability. Dentists play an important role in communicating recommendations for health behavior change to improve or maintain oral health [57, 61]. This includes giving information on regulations on co-payments and fixed allowances for dental treatments in an understandable way. It is important that co-payments for dental treatment can be financed in order to address unmet needs for dental care and eventually reduce social inequalities in oral health [59]. In Germany, people with statutory health insurance who have at least one dental check-up a year have lower co-payments. The finding that people without annual dental visit more often had reduced chewing ability leads to the question of how these people can be better reached. General practitioners could contribute to this by motivating their patients to visit the dental practice more often [56, 62]. The same applies to home care services, which should encourage and help their patients to visit the dentist regularly [56]. An expert standard for the promotion of oral health in nursing care was recently published in Germany [63]. Beneficial outcomes of home visiting programs in order to maintain health and autonomy of older individuals were reported inconsistently in literature, either reporting reduction of disability burden [64], or no health effects at all [65]. Adversely, oral health care programs may contribute to an improvement of daily activities in older patients requiring home nursing care, by recovering or maintaining dental health or occlusal support by preventing tooth loss [66, 67]. In Germany, the AuB-concept was founded in 2010 by the Federal Dental Association and the Federation of Panel Dentists [68]. This concept was the first to systematically address the care of vulnerable patient groups who usually have poorer oral health compared to the general population like older immobile individuals, and people with disabilities. This concept led to new billing codes as additional remuneration for the required outreach to insured persons by the Statutory Health Insurance Structure Act, which came into force in 2012, thus enabling professional oral health care for home care recipients or nursing home residents by dentists, working in private practices. A comprehensive community-based oral health care by public health dentists, who are working in community health authorities, is not yet a mandatory task in Germany.

A study on the relationship between childhood circumstances and chewing ability in adulthood was able to show that socioeconomic and behavioral factors in childhood have lasting effects on chewing ability in middle and later adulthood [69]. This underlines the importance of regular prevention interventions that start early to promote oral health and oral health behavior [70, 71]. Here, special attention should be paid to individuals from socially disadvantaged backgrounds because they are more likely to have an unhealthy lifestyle (e.g. daily smoking) [48] and they have a lower control-oriented dental utilization than those of middle and high SES [72, 73]. Maintaining dental health in childhood is of great importance because damage to permanent teeth is irreversible and affects oral health in all following life stages [74]. Due to their extensive preventive programs and their outreach care in nursing and elementary schools as well as in colleges, community-based Public Dental Health Services in Germany have a crucial impact concerning the amelioration of population-based dental health care measures, by empowering children to perform an adequate oral hygiene, in order to maintain their teeth healthy, independently from their social background [75]. Thus, the strengthening of Dental Public Health measures in childhood might be a useful step towards an increase in social justice and equal health opportunities in Germany, eventually benefiting all age groups into high adulthood.

The present study has some *strengths and limitations* that need to be discussed. This article is the first to comprehensively analyze the chewing ability, including associated factors, in older people based on data from a sample representative of the population in Germany. The high number of participants in GEDA 2019/2020-EHIS allows stratification according to various characteristics and thus a detailed examination of chewing ability in selected subgroups. However, participation rates in older people are lower compared to the general population, and persons affected by health limitations are less likely to participate in health surveys [76, 77]. Beyond that, people living in residential facilities or nursing homes were not included in the survey. This can result in selective non-participation and consequently under-representation and bias of the results (selection bias) [78]. Regarding the indicator on utilization, which asks about dental and orthodontic visits, it should be noted that in Germany orthodontic treatment is not a standard treatment in adulthood. Statutory health insurance covers dental visits, but orthodontic treatment only up to the age of 18 and merely from a certain degree of severity [79]. It can therefore be assumed that participants reported almost exclusively dental visits. In addition, this article was able to show that reduced chewing ability is associated with self-reported unmet needs for dental care. However, it is unclear what kind of dental treatment the respondents could not afford. This would be important information, as in Germany the amount of costs for these treatments can certainly vary [80]. Moreover, it is important to note that functional limitations such as reduced chewing ability are an integral component of the broader concept of oral health-related quality of life (OHRQoL) [81] that can be assessed, for example, with the OHIP-5 (Oral Health Impact Profile) [82]. As mentioned in the introduction, international studies indicate a strong relationship between chewing ability and OHRQoL [9, 20, 24, 27, 28]. In GEDA 2019/2020-EHIS, no information on OHRQoL is available. Therefore, corresponding analyses are still pending for Germany.

Chewing, as well as mastication, were often used as synonyms in literature for the procedure of processing food during dietary intake [83–87]. The Glossary of Prosthetic Terms defines mastication ‘as the process of chewing food for swallowing and digestion’ [88]. Based on this definition, chewing may be considered as one active element among others, which are subsumed under the umbrella term ‘mastication’. The ability to process food properly may be impaired by a multitude of parameters, including chewing, eating and saliva disorders, deterioration of oral motor skills, the oral health status, or oral pain, as published in a systematic review [89]. In this publication, masticatory dysfunction was subsumed under the generic term ‘deterioration of oral motor skills’, while chewing difficulties were classified under ‘chewing, eating and saliva disorders’, thus displaying a clear separation between the terms ‘chewing difficulties’ and ‘masticatory dysfunction’. Again, this insight may be seen as a further example for a current lack of consensus among researchers on the exact classification or use of semantics concerning the terms ‘mastication’ and ‘chewing’, as stated in literature [90]. In order to simplify the decision on the correct terminology, and due to the obviously homologous use of both terms in a certain number of publications, as well as with respect to the wording of the questioning in the survey, the term ‘chewing ability’ was used in the present publication.

## Conclusion

Based on nationwide, representative survey data, the present study is the first to examine the chewing ability, including associated factors, in older adults in Germany. Thus, this article fills a gap, as there are very few studies from Europe on this topic. According to the results, one in five adults from 55 of age had reduced chewing ability. In this respect, this is a very common functional limitation in older age. Reduced chewing ability was associated with old age, low SES, limitations to usual activities due to health problems, depressive symptoms, daily smoking, low dental utilization, and perceived unmet needs for dental care. Therefore, its prevention requires a holistic consideration of the living environment and health care context of older people. Given that chewing ability influences quality of life and social participation [2], maintaining or improving chewing ability is important for healthy aging [8].

## Data Availability

The authors state that some access restrictions apply to the data on which the results are based. The dataset cannot be made publicly available because the informed consent of the study participants does not cover the public provision of the data. The minimal dataset underlying the results is archived at the Research Data Center of the Robert Koch Institute and can be accessed by researchers upon reasonable request. Data access is available on-site at the Secure Data Center of the Robert Koch Institute Research Data Center. Requests can be made by e-mail to fdz@rki.de.

## List of abbreviations

AAPOR: American Association for Public Opinion Research
BMI: Body Mass Index
CATI: Computer Assisted Telephone Interview
DGEM: German Society for Nutritional Medicine
DSM-IV: Diagnostic and Statistical Manual of Mental Disorders
EHIS: European Health Interview Survey
GEDA: German Health Update
PHQ-8: Patient Health Questionnaire
PR: Prevalence ratios
SES: socioeconomic status
WHO: World Health Organization
95% CI: 95% confidence intervals
